# Supplementary material for: Patterns of Intron Gain and Loss in Fungi
Source: PLoS Biol. 2004 Nov 30;2(12):e422. doi: 10.1371/journal.pbio.0020422 (PMC532390; doi:10.1371/journal.pbio.0020422)
Supplement: Table S1 — Also available at http://genes.mit.edu/NielsenEtAl/. (4.3 MB ZIP). [file pbio.0020422.st001.zip › NielsenEtAl/html/807.html]

AN0762.1.NCU04124.1.MG06735.1.FG01873.1


```
 CLUSTAL W (1.82) Multiple Sequence Alignments - Introns Inserted


Sequence 1: NCU04124.1	743 aa
Sequence 2: MG06735.1	761 aa
Sequence 3: FG01873.1	722 aa
Sequence 4: AN0762.1	732 aa
Alignment Length: 792 aa
Number Identitical Residues: 314 aa
Alignment Score (without introns) 15764


MG06735.1 	-----MAEAAPSTSR---TSAGG-------TENANKFQHAISAWRT1VDLTNLVSGLDNT
NCU04124.1	-----MADVISPEAL---GAGGGGGGGGDAPNATNKFQSAISQWRS1LDFTSLVSNLDNT
FG01873.1 	-----MSDAAVEAAL---ASTPEPVAPAPTFEGENKFQHAISAWRT1IDLTTLVSNLDNT
AN0762.1  	MDSFAITEGIIPQSEKQDAQAPESGLGPALPEGANKFQRAIAAWRG1IDLSNTLAKLDST
          	 .: ::::     : ...             :  **** **: **  :*::. :: **.*

MG06735.1 	ASEIVTYQRDSVVQRKDLAQKTKDFRKLDDASKLAEIKGLLKA1YQTFIDLLTNHSKSIN
NCU04124.1	ASEIVTFQRDSTVQRKDLAQKTKDFRKLDDATKLGEIKGLLKA1YQGFIDLLTNHSKSVN
FG01873.1 	ASDIVAYQRDSTVQRKELAQKTKEFRKLDDSSKLTEIKGLLKS1YQTFIDLLTNHSKSVN
AN0762.1  	ASDIVAEQRDALVQRKDLAQKTKDFRKLDDASKLAEYKGLLKA1YQGFIDLLTNQGKSSS
          	**:**: ***: ****:******:******::** * *****: ** *******:.** .

MG06735.1 	SSFIQAYNALSEAPDPYPLLEAAVDSMLLTEDTLPKLTEENQHLQKTVTQLTSQLDDTES
NCU04124.1	SAFLHAYTSLSEAPDPYPLLEASVDSMLVSEDTLPKLTEENSHLQKNVAKLTSQLEETES
FG01873.1 	SAFLQTYASFSDAPDPYPLLEASVDSMLLAEDTLPKITEENQHLQENVTTLTTQLEDVES
AN0762.1  	SAFLQLYSSLSEAPDPYPLLEASIDSLVVAEETVPKLTSERDQLQSSVDRLTSQLEDTER
          	*:*:: * ::*:**********::**::::*:*:**:*.*..:**..*  **:**::.* 

MG06735.1 	RLQTERAARKELEDNLESRVKEVETTWIAVVDEKKDNWAAKEKALEEKLENQERLLSGIR
NCU04124.1	RLQKESDARRQLEENLESKVREVESSWNAVLEEKQDNWTAKEKALEEKIENQDRLLSELK
FG01873.1 	KLQTERKARKDLEENLEKRVKEVETSWTAVLDEKTDNWEAKEKSLEDKVEKQERLLTEMR
AN0762.1  	RLEEERAARKKLEDNQDAKIKEIETSWSAVLTEKTNNWTSKEKSLEEKVENQERLIKELK
          	:*: *  **:.**:* : :::*:*::* **: ** :** :***:**:*:*:*:**:. ::

MG06735.1 	ATEEVNERLGKSTSDADESQQRSYVMGAELEMVNSELERTSARLAEVEARNEQLRLELAE
NCU04124.1	ASYEVNQRLGKSD---DAEEQTGHVTSAELEMVQSDLERTSTRLAEVEARNEQLRLELAQ
FG01873.1 	ASYEVNQRLGKNG--EEQETQRNQVSSAELEMLHSDLDRTSARLAEIEARNEQMRLELAQ
AN0762.1  	ASYEVSQRLGQTD--ESGNHPQGGATAAELELVSSELEKTSLRLAEMEGRNEQLRLELAQ
          	*: **.:***:.    . .   . . .****:: *:*::** ****:*.****:*****:

MG06735.1 	QAKKSQSSNQLQNQQLEDDPAYMRMRSENSSLMRKLDASRVEKESLRRDLDTRMRGLERE
NCU04124.1	--SKSQVSAQP-AISLEDDPGYLRVRSENSSLIRKLDAARVDKEALKRELDAKLRGLERE
FG01873.1 	--AKSSAQQQP-ETNLEDDPGYMRVRSENQSMIRKLDAARVEKEGLKRDLDGKLRSIERE
AN0762.1  	--AVSHSKEER-TTSIDDDPGYLRLQSENSSLLRKLDAARFDRESERHTWEAKLLQSERQ
          	    *  . :    .::***.*:*::***.*::*****:*.::*. ::  : ::   **:

MG06735.1 	VALLREERENLRAKVQKWSDYEDIKQELEVLK~SIEFAMGDEDDVQGGQDDDTGADASNT
NCU04124.1	VGLLKEERDALKAKVQKWSDYDDVKQELEVLK~SIEFATGDDDDAVRAVAGDN-ADGDNE
FG01873.1 	VNALKEERDTLKNKVQKWGDYDEIKQELEVLK~SIEFSTGDDDEVREHLESKD-------
AN0762.1  	FSKVNAEKEELKTRLEKVADYEDIRRELEMIK0SIEFSAGDDEEAGDLNDGTNGTVDKAK
          	.  :. *:: *: :::* .**:::::***::* ****: **:::.     .  .:  .  

MG06735.1 	GKGKGDTLEQLLLARNKKLSDELTILRVSHQDLQTRLSDLQEELSRTNAELERSQNLNEK
NCU04124.1	ARGKGDTLEQLLLARNKKLNDELTILRVSHQDLQTRLQDLQEELSRTNAELEKSQNLNEK
FG01873.1 	--SEGNSLEKLLLARNKKLGDELTVLRVSHNDLQSRLEDLQEELSRTNAELERSQKLNQK
AN0762.1  	EGGKNGSLEQLLLARNKKLTDELTVLRVSHRDLQGQLETLREDLSTTKEELEKSQNLSTT
          	  .:..:**:********* ****:*****.*** :*. *:*:** *: ***:**:*. .

MG06735.1 	LENDLSTLQAEGANAFP-SGASVAGTYVNRYAPSMAGSRRPGAGGGRTSPTSSIISGFAS
NCU04124.1	LEDELSTIQAEAPNAFP-SGASVA-----RYAPSIAPTRK--LGGGRISPTSSIVSGFNP
FG01873.1 	LETDLETIQEEGANAFP-SGASVAG----TYVTRAVGRKS-----GRISPTSSIISGMDP
AN0762.1  	LENDLLRLQQEAANAFPSSAMSVAG----TYVSKYPHSSR-----RGVSPTSSIISGFDQ
          	** :*  :* *..****:*. ***.     *..               ******:**:  

MG06735.1 	GGGGLGSERDP-----MGGGSGILPMITAQRDRFKKRNAQLEQELSETHRNISQLRQEIA
NCU04124.1	RGADRDSPDFA-----GGGAGGILPMITAQRDRFKKRNAQLEQELSETHKTVSQLRQEIA
FG01873.1 	RMGGGEPGERV-----YGGGSGMLPMVTAQRDRYKKKNGELEEELSNTHRTVSQLRQEVA
AN0762.1  	SAASNNTMDAIRAGEAVGGGSGLLPMIQAQRDRFKKKNTELEEELSKLYSTVKSLRQEVA
          	  ..  .     :..: **..*:***: *****:**:* :**:***: : .:..****:*

MG06735.1 	ALQKDNLKLYEKSRYVQSYNRGGGMHPTAAAGASPMMGGAVSS~SSSAFSANPNPSTISI
NCU04124.1	ALQRDNLNLYEKTRYVSSYNRAGG-------------GMASAT~STASFSSNPNPSAVSF
FG01873.1 	ALQKDNLNLYEKTRYVSTYNRGGG-----------------AA~TSSAYGANPNPSTVSI
AN0762.1  	SLQKDNLNLYEKT-----------------------------S2ASSIHTSADTPS----
          	:**:***:****:                             : ::: . :  .**    

MG06735.1 	GGGSIN------GPIALDQYRKAYESNLSPFAAFRGRESARAYKRMSLPERVVYSITRMV
NCU04124.1	GATSGSDGNHGGGGVALDRYRKAYESNISPFAAFRGRESARAYKRMSLPERAVYSITRMV
FG01873.1 	GETGNP-------GIAMDRYRQAYESNISPFAAFRGRESARAYKRMSLPERAVYSLTRTV
AN0762.1  	-------------GLSIDRYQSAYEAQISPFAAFRGRESTRAYKRMSLPERVVFSLTRII
          	              :::*:*:.***:::***********:***********.*:*:** :

MG06735.1 	LASRTSRNLFAAYCLALHLLVFMSLYWLGTVDVENHASNLGGAAAAAAAAGHGAKGAAA-
NCU04124.1	LATRTSRNLFAAYCVALHVLVFLSLYWMGSADADQHVVTAAVGAVGSAAGGAGRS-----
FG01873.1 	LASRTSRNLFGAYCVALHLLVFMCLYWMSSSDVQRVTHLESAAAAAAGVAGGSLG-----
AN0762.1  	LANRTSRNLFAGYCFALHILLFIMLYMMSTMEIESHSAASLGAAAGAAMNAAGNGNAYSG
          	**.*******..**.***:*:*: ** :.: : :        .*..:.  . .  .: :.

MG06735.1 	--AHGDWHSDDFKGT-
NCU04124.1	--SGGEWSEEGFSHR-
FG01873.1 	--SPMDGAPKDPAK--
AN0762.1  	QLDGDDWQQEGFNHAG
          	.    :   ..    .
```
